# *Circ*-*Foxo3* is positively associated with the *Foxo3* gene and leads to better prognosis of acute myeloid leukemia patients

**DOI:** 10.1186/s12885-019-5967-8

**Published:** 2019-09-18

**Authors:** Jiao Zhou, Ling-Yu Zhou, Xi Tang, Jing Zhang, Ling-Ling Zhai, Yun Yun Yi, Jing Yi, Jiang Lin, Jun Qian, Zhao-Qun Deng

**Affiliations:** 1 Department of Laboratory Center, The Affiliated People’s Hospital of Jiangsu University, 8 Dianli Rd, Zhenjiang, 212002 Jiangsu People’s Republic of China; 2 The Key Lab of Precision Diagnosis and Treatment of Zhenjiang City, Zhenjiang, Jiangsu People’s Republic of China; 3 Department of Hematology, The Affiliated People’s Hospital of Jiangsu University, Zhenjiang, 212002 Jiangsu People’s Republic of China

**Keywords:** *Foxo3*, Circular RNA, acute myeloid leukemia, Better prognosis

## Abstract

**Background:**

The *Foxo3* gene, belonging to the forkhead family, is one of the classes of transcription factors characterized by a forkhead DNA-binding domain, which usually considered being a cancer suppressor gene. *Circ-Foxo3* is a circular structure which connects the 3’end to the 5’end. Scholars detected that *circ-Foxo3* could compete with *Foxo3* for binding to some miRNAs.

**Methods:**

In this study, we will test the expression of *Foxo3* and *circ-Foxo3* in de novo acute myeloid leukemia (AML) patients to explore the relationship between *Foxo3* gene and *circ-Foxo3*. All the de novo AML samples and normal control samples was measured by real-time quantitative PCR. A receiver operating characteristic curve was conducted to differentiate AML patients from control people. Association of *Foxo3* expression and overall survival was conducted by Kaplan-Meier survival analysis.

**Results:**

We found that the expression of *Foxo3* gene in de novo patients was significantly lower than control samples (*P* = 0.009). Meanwhile, *circ-Foxo3* also expressed lower in de novo AML patients than in control samples (*P* = 0.040). In different classifications, this trend could be observed more remarkably. In non-M3 patients, the *Foxo3* high patients’ survival time was longer than *Foxo3* low patients (*P* = 0.002). Besides, in non-favorable risk groups, patients with low expression of *Foxo3* had longer survival time than *Foxo3* high patients (*P* = 0.004). Furthermore, in normal Karyotypic patients, the overall survival time of patients with high-expressed *Foxo3* was significantly longer than those with low expression (*P* = 0.034). Besides, Pearson analysis was also conducted between these two genes in AML patients. Results revealed that they were positively correlated (R = 0.63, *P* < 0.001).

**Conclusion:**

In conclusion, we found that low expression of *circ-Foxo3* and *Foxo3* were frequent in AML patients, and patients with high expression of *Foxo3* often had a trend of better prognosis.

## Background

Acute myeloid leukemia (AML) is one of the most common types of hematological neoplasms in both child and adult. It is acknowledged that AML is a malignant clonal disease which results from blocked differentiation and uncontrolled proliferation or accumulation of abnormal hematopoietic cells. It is diagnosed mainly on the morphological examination of bone marrow and peripheral blood. Specific diagnosis is confirmed by immunophenotyping and cytochemistry searching for myeloperoxidase activity in blasts, or by immunophenotyping surface type molecules [1]. The France- America– British classification (FAB classification), by the degree of differentiation and morphology, divides AML patients into eight subgroups (M_0_-M_7_) [2]. Besides, on the basis of World Health Organization (WHO) criteria, according to different chromosome types and genotypes, AML can be divided into three different prognostic groups, including favorable, intermediate and poor. In favorable group, genetic abnormalities often include t (8;21), t (15;17),inv.(16), normal karyotype with *NPM1* mutations but in absence of *FLT3-ITD* or isolated biallelic; in adverse group, it includes inv. (3), t (3;3), t (6;9),-5,5q-,-7,7q-, complex karyotype or normal karyotype with *FLT3-ITD* mutations or TP53 mutation (Table.1); However, the prognosis of AML patients is variable due to the different clinical, pathological, molecular and genetic characteristics, including age, sex, white blood cell (WBC) count, blast, gene mutations, karyotypes, etc. [3, 4]. Therefore, it made sense to do researches on them which may change the final outcome of AML patients in the future.

**Table 1:** Definition of AML risk classification

Risk	Cytogenetic	Molecular abnormality
Favorable	Core binding factor: inv.(16) or t(16;16) or t(8;21) t(15;17)	Normal cytogenetic: NPM1 mutation in the absence of LT3-ITD, or isolated biallelic CEBPA mutation
Intermediate	Normal cytogenetic + 8 alone t(9;11) Other non-defined	
Poor	Complex (≥3 clonal chromosomal abnormalities) Monosomal karyotype −5, 5q-, −7, 7q- 11q23 - non t(9;11) inv.(3), t(3;3) t(6;9) t(9;22)	Normal cytogenetic: with FLT3-ITD mutation6, TP53 mutation

The *Foxo3* gene, belonging to the forkhead family, is one of the classes of transcription factors characterized by a forkhead DNA-binding domain. This family includes four genes: *Foxo1*, *Foxo3*, *Foxo4* and *Foxo6*, but the *Foxo3* was widely studied. It was a key regulator in the insulin/insulin-like growth factor-1signaling pathway [5–7]. It can promote people’s health through regulating stress resistance, metabolism, cell cycle and cell apoptosis. Meanwhile, it could make positive response to environmental stimuli and prevent people from suffering the disease which related to aging, such as cancers, cardiovascular disease (CVD) etc. [5, 8]. Researchers considered it as a longevity gene [9].

In cancer development, it has been supervised that elevation of AKT activity or deficiency of PTEN often resulted in down-regulation of *Foxo3*, and accelerated the formation of tumors [10, 11]. Therefore, *Foxo3* gene was taken as a tumor suppressor gene.

Circular RNA (circRNA), one of the non-coding RNAs, is a circular structure that connects the 3′ end to the 5’end [12]. It was first observed in the cytoplasm of eukaryotic through electron microscopic in 1979 [13]. In 2012, after a statistical analysis of RNA-Seq data and subsequent biochemical analysis, Salzman J. et al. found that circRNA molecules transcribed and spliced from exons in protein and noncoding genes were ubiquitous in the human and mouse genomes [14]. Moreover, studies also reviewed that the copy number of circRNA was 10 times larger than that of linear RNAs. So, they inferred that this was not a coincidence but a potential biological function in cells. Whereas, some studies have shown that some circRNA had multiple binding sites of microRNAs (miRNAs), sponging microRNA, and served as a competitive inhibitor for microRNA [15]. For example, the *circ-ITCH,* containing binding sites for miR-7, miR-17 and miR-214, inhibited the activity of these microRNAs. In 2015, a research group in Toronto found a circRNA called *circ-Foxo3*, and it promoted cell apoptosis through p53 and puma signal pathway. Scholars detected that ectopic-expressed *circ-Foxo3* could compete with some miRNAs and next adjust *Foxo3* expression [16]. It was widely accepted that the post-transcriptional repression of *Foxo3* expression was regulated by both circRNAs and microRNAs [11, 17].

In this study, we tested the expression of *Foxo3* and *circ-Foxo3* in de novo AML patients, and conducted survival analysis on the expression level and prognosis.

## Methods

### Patients and samples

The bone marrow (BM) samples of experimental group were collected from patients in the Affiliated Hospital of Jiangsu University who were initially diagnosed with acute myeloid leukemia, while the control group were from healthy BM donors or patients with chest trauma. We tested *Foxo3* gene in 122 de novo AML patients and 30 control samples. Because some of these samples had run out and new samples were collected, the *circ-Foxo3* gene was tested in 116 de novo AML patients and 24 control samples. These patients were accepted standard treatment after diagnosis of AML. The AML patients were classified by FAB classification and the 2008 WHO criteria. The therapeutic regimen and results of laboratory and equipment inspection were recorded in corresponding physician’s order sheet and medical record. All the BM donators had signed the informed consents. This study was approved by the Review Committee of the Ethics Department of the People’s Hospital of Jiangsu University.

### Treatment protocol of different patients

The patients, enrolled in this study were all received chemotherapy. The chemotherapy regimens for these patients were selected according to the NCCN (2016) guideline of AML. Patients with acute promylocytic leukemia (APL) can be divided into high risk (WBC count > WBC ≥ 10 × 10^9^/L) and low risk (WBC count≤10 × 10^9^/L) groups. For high risk patients, induction therapy was consisted of oral ATRA 45 mg/m^2^ in divided doses until clinical remission, intravenous daunorubicin 50 mg/m^2^ × 4 days, and cytarabine 200 mg/m^2^ × 7 days. After count recovery, patients received 3 monthly consolidation courses: arsenic trioxide 0.15 mg/kg·day × 5 days per week, for 5 weeks and for 2 cycles, then ATRA 45 mg/m^2^ × 7 days, with daunorubicin 50 mg/m^2^ × 3 days for 2 cycles. For low risk patients, the induction therapy was consisted of oral ATRA 45 mg/m^2^ in divided doses until clinical remission daily and intravenous arsenic trioxide 0.15 mg/kg daily until bone marrow remission. At count recovery, proceed with consolidation courses: intravenous Arsenic trioxide 0.15 mg/kg·day × 5 days per week for 4 weeks every 8 weeks for a total of 4 cycles, and ATRA 45 mg/m^2^·day for 2 weeks every 4 weeks for a total of 7 cycles [18].

For non-APL patients, who were below 60 years old, induction therapy for them consisted of 4 treatment options: a. Standard-dose cytarabine 100–200 mg/m^2^ continuous infusion × 7 days with idarubicin 12 mg/m^2^ or daunorubicin 60–90 mg/m^2^ × 3 days (for patient ≤45 y). b. Standard-dose cytarabine 200 mg/m^2^ continuous infusion × 7 days with daunorubicin 60 mg/m^2^ × 3 days and cladribine 5 mg/m^2^ × 5 days (for other age groups). c. High-dose cytarabine (HiDAC) 2 g/m^2^ every 12 h × 6 days or 3 g/m^2^ every 12 h × 4 days with idarubicin 12 mg/m^2^ or daunorubicin 60 mg/m^2^ × 3 days. d. Fludarabine 30 mg/m2 IV days 2–6, cytarabine 2 g/m^2^ over 4 h starting 4 h after fludarabine on days 2–6, idarubicin 8 mg/m^2^ IV days 4–6, and G-CSF SC daily days 1–7 [18].

For patients over 60 years old, there are options: a. Standard-dose cytarabine (100–200 mg/m^2^ continuous infusion × 7 days) with idarubicin 12 mg/m^2^ or daunorubicin 60–90 mg/m^2^ × 3 days or mitoxantrone 12 mg/m^2^ × 3 days. b. Lower intensity therapy: low-dose cytarabine or 5-azacytidine, decitabine [18].

### Definition of AML risk classification [18]

### Cell lines and cell culture

In our laboratory, human hematological cell lines (K562, U937, NB4, SHI-1, and HEL) were purchased from American Type Culture Collection Manassas, VA, USA. The cell line information can be queried from two databases: American Type Culture Collection (ATCC) and Deutsche Sammlung von Mikroorganismen und Zellkulturen (DSMZ). The ATCC number of K562, U937 and HEL were CCL-243, CRL-1593.2 and TIB-180. Details can be found on ATCC Official website. The number of SHI-1 and NB4 were ACC 645 and ACC 207, and details can be queried on DSMZ official website. The use of cell lines was approved by the Review Committee of the Ethics Department of the People's Hospital of Jiangsu University. These cell lines were cultured in RPMI-1640. The mycoplasma contamination of cell lines was negative by using quick mycoplasma test kit. Before we conducted this subject, these cell lines were tested by PCR.

### RNA isolation, reverse transcription and real time quantitative PCR

The mononuclear cells of AML patients and healthy donors were isolated from bone marrow (BMMC) using the Ficoll-Hypaque gradient. According to the manufacturer’s instructions, Trizol reagent (Invitrogen, Carlsbad, CA, USA) was employed to extract total RNA from BMMC and leukemia cell lines. The cDNA was composed through reverse transcription on the iCycler Thermal Cycler (Eppendorf, Hamburg, Germany) using a reaction mixture, which contains 2 μg of total RNA, dNTPs 10 mM, random hexamers 10 μM, RNAsin 80 units, and 200 units of MMLV reverse transcriptase (MBI Fermentas, Hanover, USA). The system of reverse transcription was incubated for 10 min at 25 °C, 60 min at 42 °C, and then stored at − 20 °C. The primers of *Foxo3* were 5′- GCAAGAGCTCTTGGTGGATCATCAA-3′ (forward) and 5′- TGGGGCTGCCAGGCCACTTGGAGAG-3′ (reverse), and the primers of *circ-Foxo3* were 5′-GTGGGGAACTTCACTGGTGCTAAG-3′ (forward) and 5′-GGGTTGATGATCCACCAAGAGCTCTT-3′ (reverse)*.* A 20 μL volume of reaction system (20 ng of cDNA, 0.8 μM of primers, 10 μM AceQTMqPCR SYBR Green Master Mix (Takara Shuzo Co, Ltd., Nojihigashi 7–4-38,Kusatsu,Shiga,Japan) and 0.4 μM ROX Reference Dye1(Invitrogen).) was used to perform Real-time RT-PCR. Amplification for *circ-Foxo3* was carried out at 95 °C for 30 min, followed by 45 cycles at 95 °C for 5 s, 68.7 °C for 30 s 72 °C for 30 s and 80 °C for 31 s. Meanwhile, amplification for *Foxo3* was carried out at 95 °C for 30 min, followed by 45 cycles at 95 °C for 5 s, 63.6 °C for 30s 72 °C for 30 s and 80 °C for 31 s. All reactions were performed on a 7500 Thermo cycler (Applied Biosystems, CA, and USA). Positive and negative controls were included in all tests. Following real-time RT-PCR, a melting curve analysis was carried out to demonstrate the specificity of the PCR product as a single peak. The relative levels of *Foxo3* transcript were calculated by the following equation: N_*Foxo3*_ = (E_*Foxo3*_) ^ΔCT *Foxo3* (control-sample)^ ÷ (E_ABL_) ^ΔCT ABL (control-sample)^. The parameter efficiency (E) was counted by the formula E = 10^(− 1/slope) (the slope referred to CT versus cDNA concentration plot)^.

*Foxo3* and *circ-Foxo3* were detected by high-resolution melting analysis (HRMA) as reported previously. DNA direct sequencing was used for confirming positive samples.

### Method for gene mutations

We tested the gene mutation (*CEBPA, NPM1, FLT3*-ITD, *KIT, N/K-RAS* and *IDH1/2*) by next-generation sequencing technology.

### Statistical analysis

All the data analysis was conducted on SPSS 20.0 software package (SPSS, Chicago, IL). Pearson chi-square analysis or variance test was used to distinguish differences in categorical variables, and the differences between two groups of continuous variables were compared by Mann-Whitney U test. In order to make a difference between the expression of AML patients and controls, Receiver operating characteristic curve (ROC) and area under the ROC curve were performed. We plotted ROC curves of AML and healthy people, and next a table of sensitivity and (1- specificity) of *circ-Foxo3* and *Foxo3* expression was drawn. We used these data to calculating the Yoden index (sensieivity+specificity-1) which could indicate the capacity of true patients and non-patients. The number which corresponding to the largest Yoden index was chosen as the cut-off value to separate AML patients into high and low groups. We used COX regression model, also known as the “proportional hazards model” (Cox model), to test if some common factors had effect on survival outcome, and if they were independent variables, This statistical method can simultaneously analyze the influence of many factors on the survival period. The impact of different *Foxo3* and *circ-Foxo3* expression on overall survival (OS) of AML patients was analyzed by Kaplan-Meier analysis. For all analyses, two-tailed *P*-values of 0.05 or less were determined statistically significant.

## Results

### Foxo3 and circ-Foxo3 expression in normal controls and de novo AML patients

**Fig. 1:**
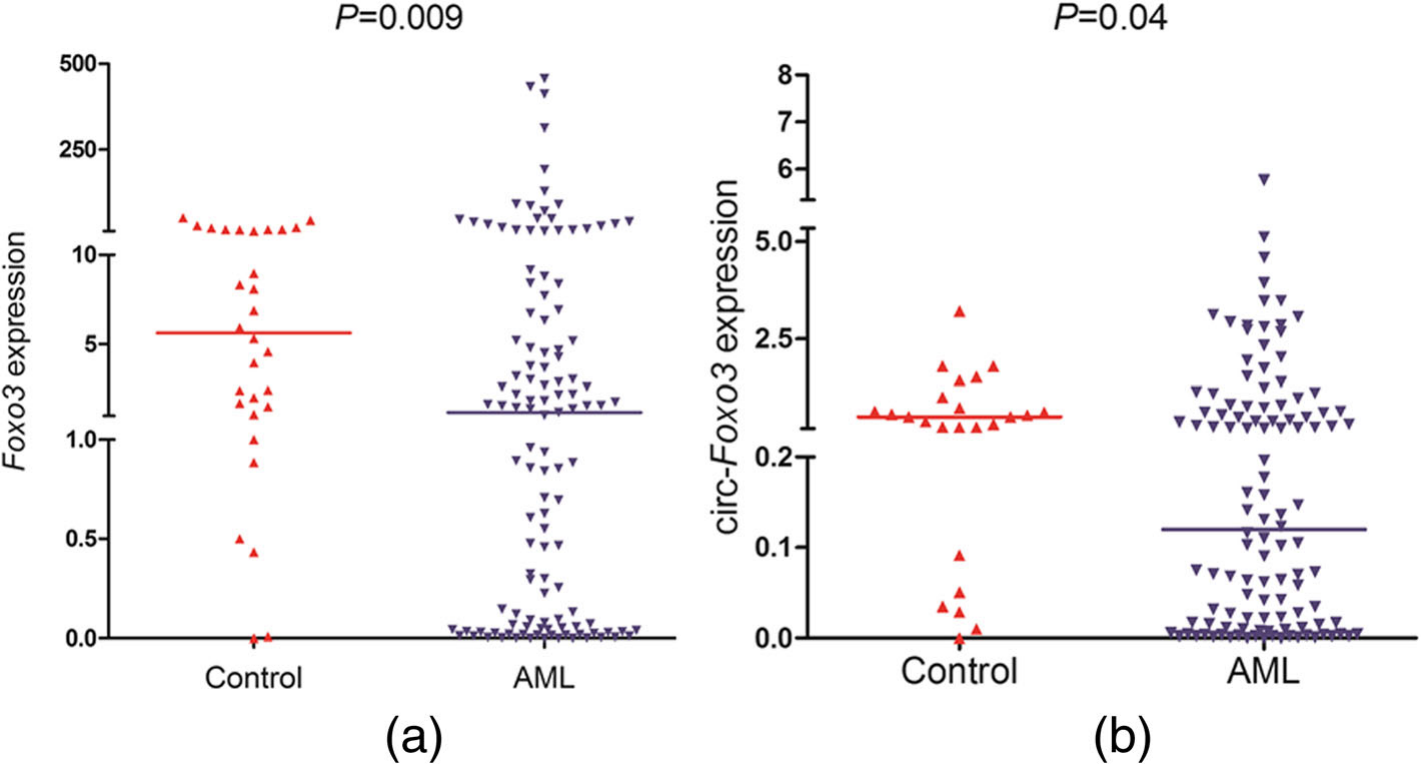
Expression of *Foxo3* and circ-*Foxo3* in BMNCs was measured via using RQ-PCR in healthy controls and the whole AML samples. Horizontal lines represent the median, and each dot represents an individual sample. Statistical analysis was performed using Wilcoxon tests, and significance was defined as *P* < 0.05

In this study, we test the expression level of *Foxo3* and *circ-Foxo3* in control people and de novo AML patients. The *Foxo3* expression level (1.0 × 10^− 6^-456.234, median 1.193) in de novo AML patients was obviously lower than in control people (0.001–49.528, median 5.619) (*P* = 0.009). Meanwhile, the *circ-Foxo3* expression level (2.8 × 10^− 5^-5.761, median 0.1198) was also lower in de novo AML patients than in control people (1.2 × 10^− 5^-3.210, median 0.5017) (*P* = 0.04). The entire scatter diagram was represented at Fig. 1.

### The diagnostic value of Foxo3 and circ-Foxo3 expression

**Fig. 2:**
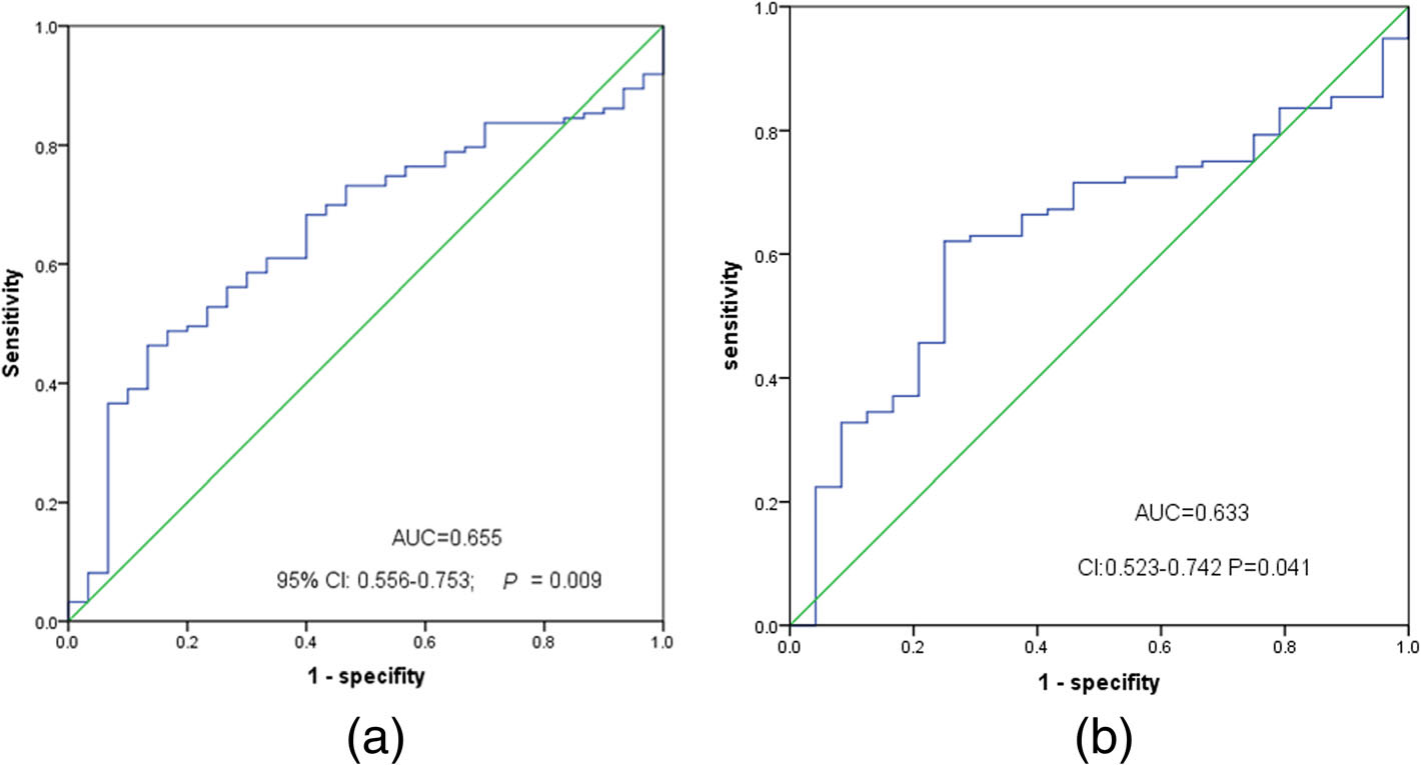
*Foxo3* and *circ-Foxo3* expression offers diagnostic tool in identification of AML patients. **a** *Foxo3* in AML patients; ROC analysis showed that the area under the curve (AUC) of *Foxo3* was 0.655 (*P* = 0.009). **b** *circ-Foxo3* in AML patients; ROC analysis showed that the area under the curve (AUC) of *Foxo3* was 0.633 (*P* = 0.041)

The diagnostic value of *Foxo3* and *circ-Foxo3* expression was analyzed by ROC curve. AML can be remarkably distinguished from controls with an AUC of 0.655 (95% CI: 0.556–0.753; *P* = 0.009) (Fig. 2a). According to the result of ROC curve analysis, we determined that the cut-off value of *Foxo3* expression was at 0.856, the sensitivity and the specificity were 44.7 and 87.7%, respectively. Similarly, the *circ-Foxo3* could differentiate AML patients from control people with an AUC of 0.633 (95% CI: 0.523–0.746; *P* = 0.041) (Fig. 2b). In a similar way, the cut-off value of *circ-Foxo3* expression was at 0.233, the sensitivity and the specificity were 62.1 and 75%, respectively.

### The correlation between Foxo3 and circ-Foxo3

**Fig. 3:**
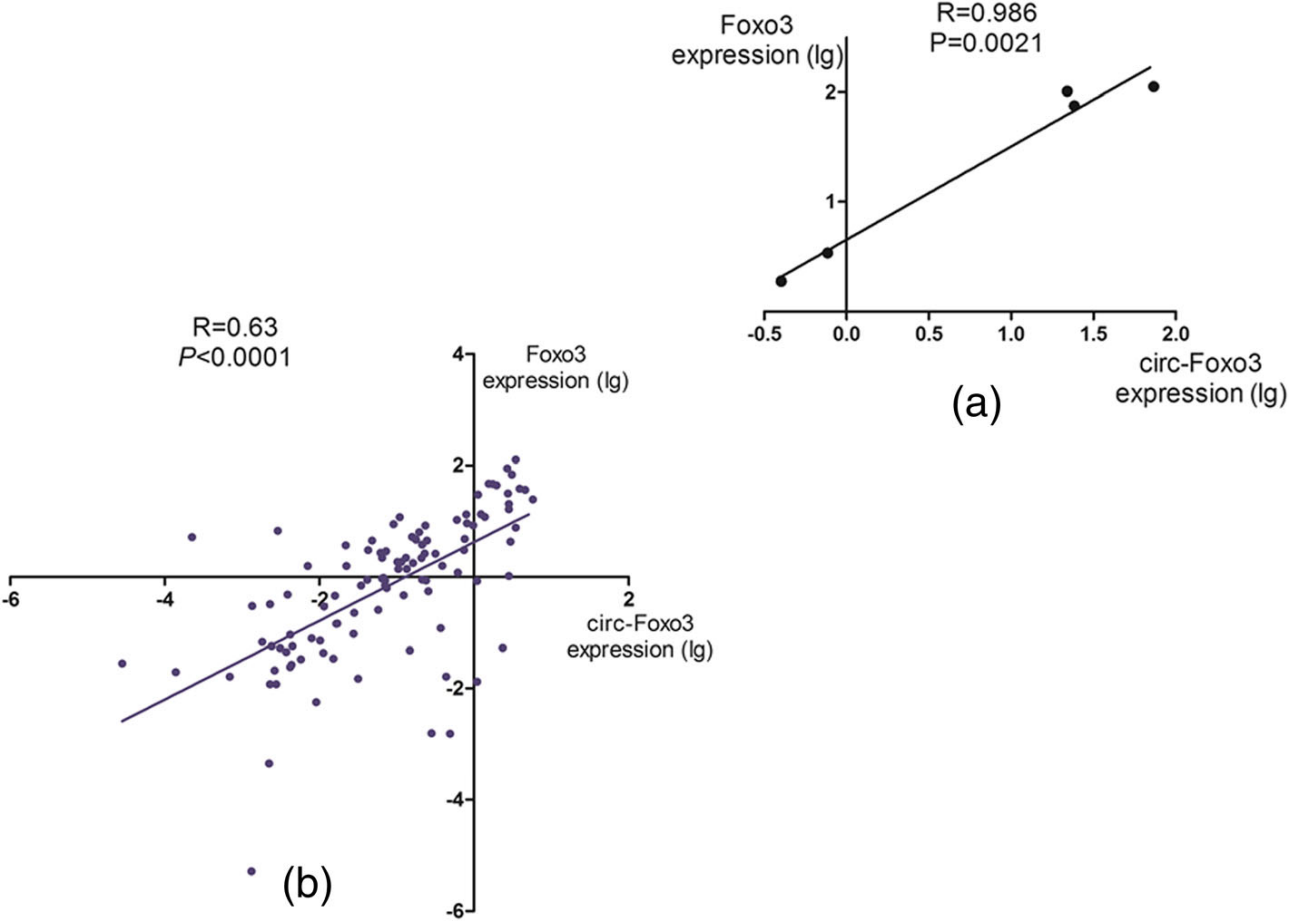
The correlation between *Foxo3* and *circ-Foxo3* expression in cell lines and AML patients was conducted on Spearman correlation test. **a** In cell lines, *Foxo3* and *circ-Foxo3* were positively correlated(R = 0.986 *P* = 0.0021). **b** In AML patients, *Foxo3* and *circ-Foxo3* were positively correlated (R = 0.63 *P* < 0.001)

In order to analysis the correlation of *Foxo3* and *circ-Foxo3*, Pearson analysis was conducted on the expression of *Foxo3* and *circ-Foxo3* in cell lines (K562, U937, NB4, SHI-1, and HEL). The result showed that *Foxo3* and *circ-Foxo3* were positively corrected (R = 0.98, *P* < 0.0021) (Fig. 3a). Besides, Pearson analysis was also conducted between these two genes in AML patients. Results revealed that they are positively corrected as well (R = 0.63, *P* < 0.0001) (Fig. 3b).

### Clinical and laboratory characteristics of AML

Based on the cut-off value of 0.856 of *Foxo3* expression, AML patients was divided into two groups, high expression group (*Foxo3* ^high^) and low expression group (*Foxo3* ^low^). The high level was over 0.856 while the low expression was below. In the same way, according to the expression of *circ-Foxo3*, at the cut-off value of 0.233, the patients were also separated into two groups, *circ-Foxo3* ^high^ and *circ-Foxo3* ^low^ group.

After analyzing the clinical data, it displayed that no statistical significance were exhibited in sex, age, white blood cells (WBC), hemoglobin (HB), platelets (PLT), BM blast, Karyotype classification, WHO classification and other seven gene mutations (*CEBPA, NPM1, FLT3-ITD, N/K-RAS, IDH1/2, DNMT3A, U2AF1* etc.) between *Foxo3* ^high^ and *Foxo3* ^low^ groups (Table.2).

**Table 2:** Comparison of clinical manifestations between AML patients and *Foxo3* expression

Patient’s parameters	High (*n* = 66)	Low (*n* = 56)	*P* value
Sex, male/female	40/26	34/22	1.0
Median age, years (range)	59 (15–87)	54.5 (10–93)	0.128
Median WBC, ×10^9^/L (range)	14.5 (0.8–528)	26.75 (0.3–197)	0.313
Median hemoglobin, g/L (range)	74.5 (34–138)	78.5 (49–135)	0.134
Median platelets, ×10^9^/L (range)	41 (3–264)	39.5 (5–415)	0.921
BM blasts, % (range)	46.25 (5.5–99)	42 (1–94.5)	0.197
CR (−/+)	33/30	29/24	0.853
FAB classification			0.428
M_0_	1 (1.5%)	0 (0%)	
M_1_	6 (9.1%)	2 (3.6%)	
M_2_	29 (43.9%)	19 (33.9%)	
M_3_	8 (12.2%)	15 (26.8%)	
M_4_	13 (19.7%)	12 (21.4%)	
M_5_	8 (12.1%)	7 (12.5%)	
M_6_	1 (1.5%)	1 (1.8%)	
Karyotype classification			0.666
Favorable	18 (27.3%)	19 (33.9%)	
Intermediate	37 (56.1%)	28 (50%)	
Adverse	8 (12.1%)	7 (12.5%)	
No data	3 (4.5%)	2 (3.6%)	
Karyotype			0.423
normal	27 (40.9%)	20 (35.7%)	
t(8;21)	8 (12.1%)	4 (7.1%)	
t(15;17)	9 (13.6%)	15 (26.8%)	
11q23	0 (0)	0 (0)	
complex	8 (12.1%)	5 (8.9%)	
others	11 (15.2%)	10 (30.4%)	
No data	3 (4.5%)	2 (3.6%)	
Gene mutation			
*CEBPA* (+/−)	10/51	3/41	0.229
*NPM1* (+/−)	4/57	3/41	1.00
*FLT3*-ITD (+/−)	7/54	5/39	1.00
*KIT* (+/−)	2/59	2/42	1.00
*N/K-RAS* (+/−)	8/53	2/42	0.187
*IDH1/2* (+/−)	2/59	2/42	1.00
*DNMT3A* (+/−)	4/57	5/39	0.487
*U2AF1* (+/−)	2/59	1/43	1.00

*WBC* white blood cells, *FAB* French-American-British classification, *AML* acute myeloid leukemia, *CR* complete remission. Percentage was equal to the number of mutated patients divided by total cases in each group

### Correlation between Foxo3 expression and clinical outcome

**Fig. 4:**
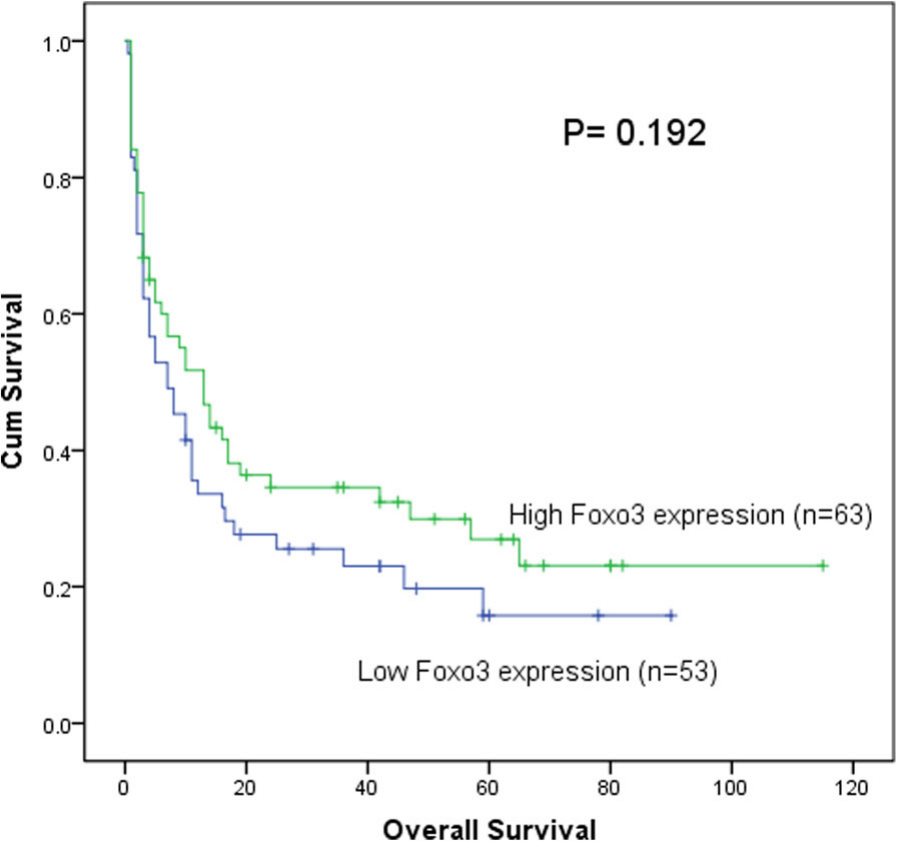
The impact of *Foxo3* expression on overall survival of the in AML patients. Survival analysis was performed via Kaplan–Meier survival analysis, with differences between curves analyzed via a log-rank test(*P* = 0.192). Significance was defined as *P* < 0. 05

**Fig. 5:**
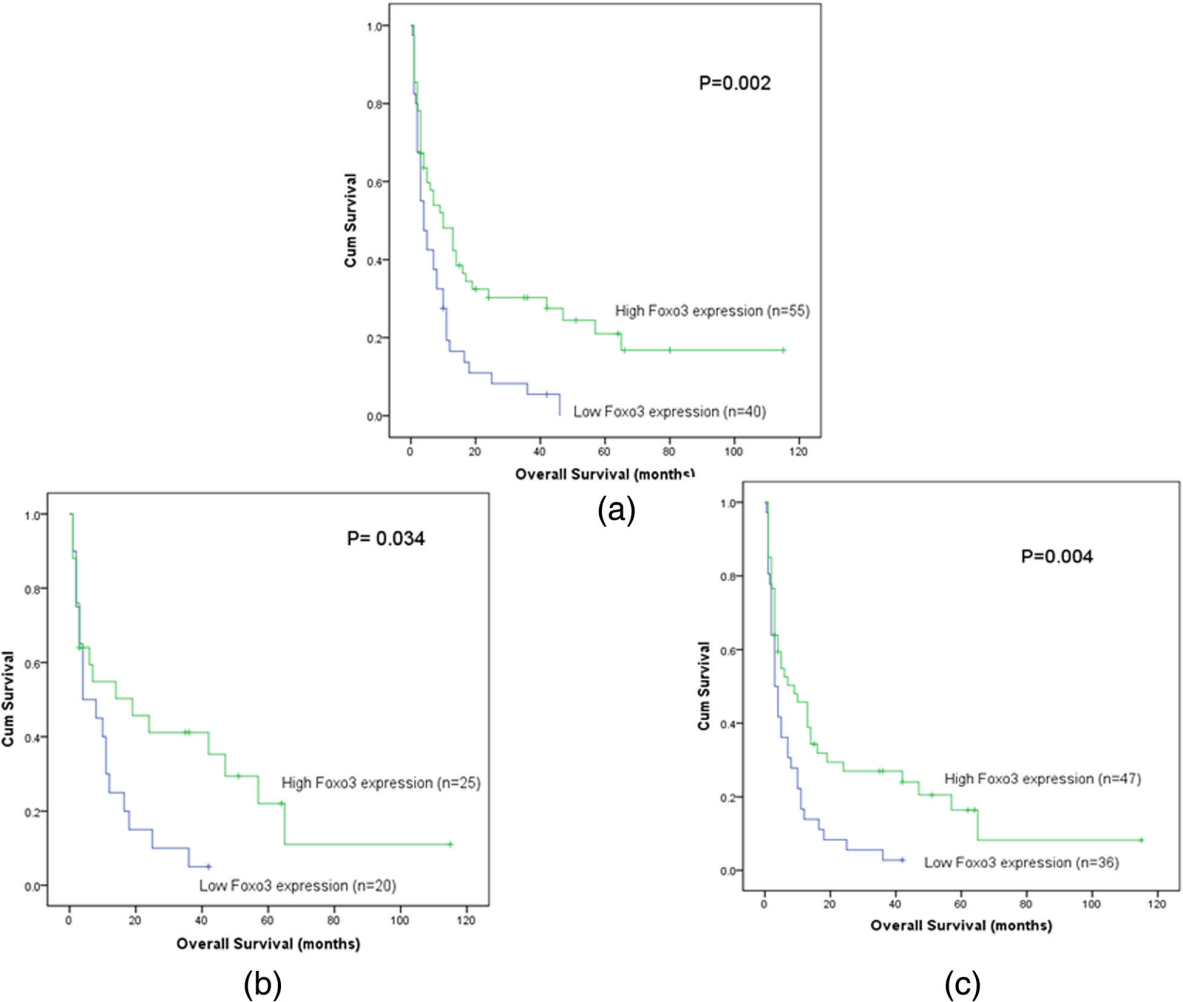
The impact of *Foxo3* expression on overall survival of the different groups of AML patients. **a** non-M3 patients. **b** Normal Karyotypic patients. **c** Non-Favorable patients. Survival analysis was performed via Kaplan–Meier survival analysis, with differences between curves analyzed via a log-rank test. Significance was defined as *P* < 0. 05

**Fig. 6:**
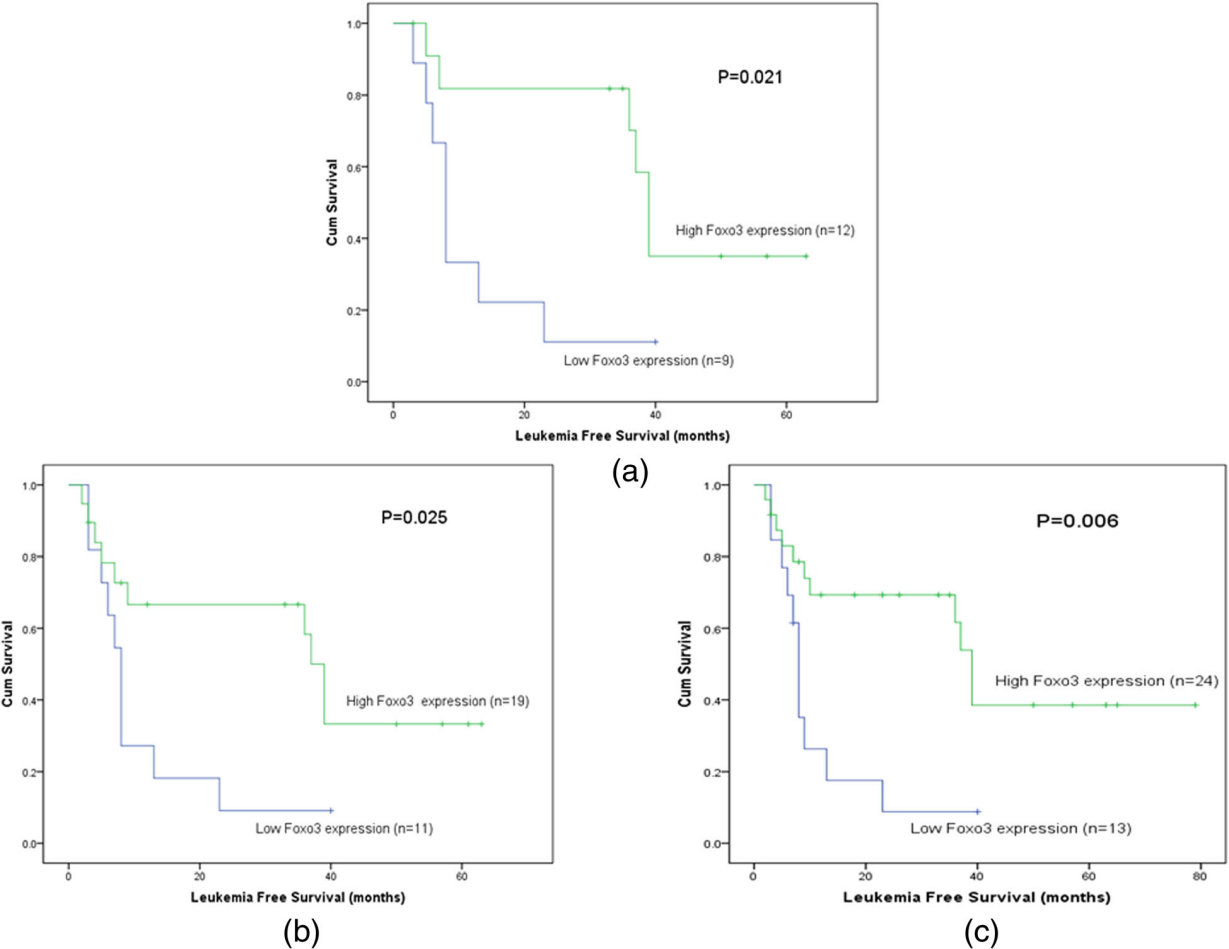
The impact of *Foxo3* expression on leukemia-free survival time of the AML patients. **a** Normal Karyotypic patients. **b** Non-Favorable patients. **c** non-M3 patients

In this study, total median follow-up time of the patients we tested the *Foxo3* expression was 8.5 months. We carried out Kaplan-Meier survival analysis on *Foxo3* ^high^ and *Foxo3* ^low^ patients. It was found that there was a trend that *Foxo3* ^high^ patients’ survival time (95% CI, 15.75–29.42 months, median value, 10 months) was longer than *Foxo3* ^low^ group (95% CI, 10.54–22.46 months, median value, 7 months) (*P* = 0.192) (Fig. 4). In different classifications, this trend could be observed as well. In patients without M3 patients (non-M_3_ patients), the *Foxo3* ^high^ patients’ OS time (95% CI, 12.90–26.74 months, median value, 9 months) was longer than *Foxo3* ^low^ patients (95% CI, 5.09–12.08 months, median value, 4 months) (*P* = 0.002) (Fig. 5a). Besides, in normal Karyotypic patients, the overall survival time of *Foxo3* ^high^ patients (95% CI, 12.23–36.33 months, median value, 7 months) was significantly longer than *Foxo3* ^low^ patients (95% CI, 5.31–16.24 months, median value, 6 months) (*P* = 0.034) (Fig. 5b). Furthermore, in intermediate and adverse (non-favorable) risk groups, *Foxo3* ^low^ patients (95% CI, 4.22–10.69 months, median value, 3.5 months) had shorter survival time than *Foxo3* ^high^ patients (95% CI, 10.91–25.13 months, median value, 6 months) (*P* = 0.004) (Fig. 5c). In the same way, the leukemia free survival time (LFS) of different classifications of patients was also analyzed. We found that in non-M3, non-favorable and normal Karyotypic groups, patients with high level of *Foxo3* expression had longer LFS time than low level patients (Fig. 6).

Univariate and multivariate analyses (COX regression Model) were also conducted, applying age (≤ 60 y vs. > 60 y), sex (male vs. female), WBC (≥ 30 × 10^9^/L vs. < 30 × 10^9^/L), HB (< 110 g/L vs. ≥110 g/L), PLT (100 × 10^9^/L vs. 100 × 10^9^/L), karyotype classifications (favorable vs. intermediate vs. poor), gene mutations (mutant vs. wild-type) and *Foxo3* expression status (high vs. low) as covariates. The univariate analysis showed that NPM1, Age, Karyotypic classification and WBC count were independent risk factors of AML patients (Exp > 1). After multivariable analysis, it figured out that Karyotypic classification, *Foxo3* expression and Age were factors that affected AML prognosis. Among them, *Foxo3* expression was a protective factor. Besides, Karyotypic classification and age were adverse prognosis factors either (Exp < 1) (Table.3).

**Table 3:** Univariate and multivariate analyses of prognostic factors for overall survival in AML patients

Whole patients	Univariate			Multivariate		
	HR (95% CI)	Exp.	*P* value	HR (95% CI)	Exp.	*P* value
*Flt3-ITD*	0.575–2.330	1.158	0.681	0.885–2.330	1.435	0.295
*NPM1*	1.044–5.017	2.289	0.039	0.471–3.644	1.309	0.606
*CEBPA*	0.515–2.253	1.077	0.843	0.402–3.666	1.215	0.730
WBC count	1.891–4.505	2.918	< 0.001	0.804–2.571	1.437	0.221
Karyotypic classification	1.628–2.763	2.121	< 0.001	1.113–2.188	1.561	0.010
*Foxo3* expression	0.499–1.156	0.762	0.210	0.341–0.951	0.569	0.031
Circ-*Foxo3* expression	0.650–1.661	1.039	0.873	0.664–2.200	1.208	0.536
Age	2.274–5.538	3.549	< 0.001	1.854–5.523	3.200	< 0.001

### Correlation between circ-Foxo3 expression and clinical outcome

We also studied the clinical data of patients with high and low level of *circ-Foxo3* expression. Results showed that patients with high level of *circ-Foxo3* expression survived longer (95% CI, 11.28–25.16 months, median value, 8 months) than low level of that (95% CI, 11.93–24.07 months, median value, 7 months), although this was out of statistical significance (*P* = 0.762). More than that, the difference of overall survival time between high and low level of *circ-Foxo3* expression in different patients group also had no statistical significance. Multivariate analysis (COX regression model) was also conducted, applying age (≤ 60 y vs. > 60 y), sex (male vs. female), WBC (≥ 30 × 10^9^/L vs. < 30 × 10^9^/L), HB (< 110 g/L vs. ≥110 g/L), PLT (100 × 10^9^/L vs. 100 × 10^9^/L), karyotype classifications (favorable vs. intermediate vs. poor), gene mutations (mutant vs. wild-type) and *Foxo3* expression status (high vs. low) as covariates. The result demonstrates that, in our study, these covariates above did not affect the prognosis of patients.

## Discussion

Both circular *Foxo3* (*circ-Foxo3*) and linear *Foxo3* (*Foxo3* mRNA) are encoded by *Foxo3* gene [19]. Recently, *Foxo3* gene was widely believed to be a tumor suppressor gene in many cancers, like breast [20, 21] and ovarian [22] cancer. Besides, *circ-Foxo3* was found to down-regulated in patient tumor samples and in a group of cancer cells [20]. Currently, AML has been internationally recognized to be a malignant clonal disease with multiple prognoses: 5-year overall survival was less than 50%, and only 20% of older patients will survive within 2 years of diagnosis [2, 23] while there are many treatment programs for this. Associated with karyotypes and molecular mutations, AML patients can be assorted into diverse prognostic risk groups [24]. To investigate whether *Foxo3* and *circ-Foxo3* were two factors which affect the prognosis of AML, the expression of *Foxo3* and *circ-Foxo3* was tested in AML patients.

**Fig. 7:**
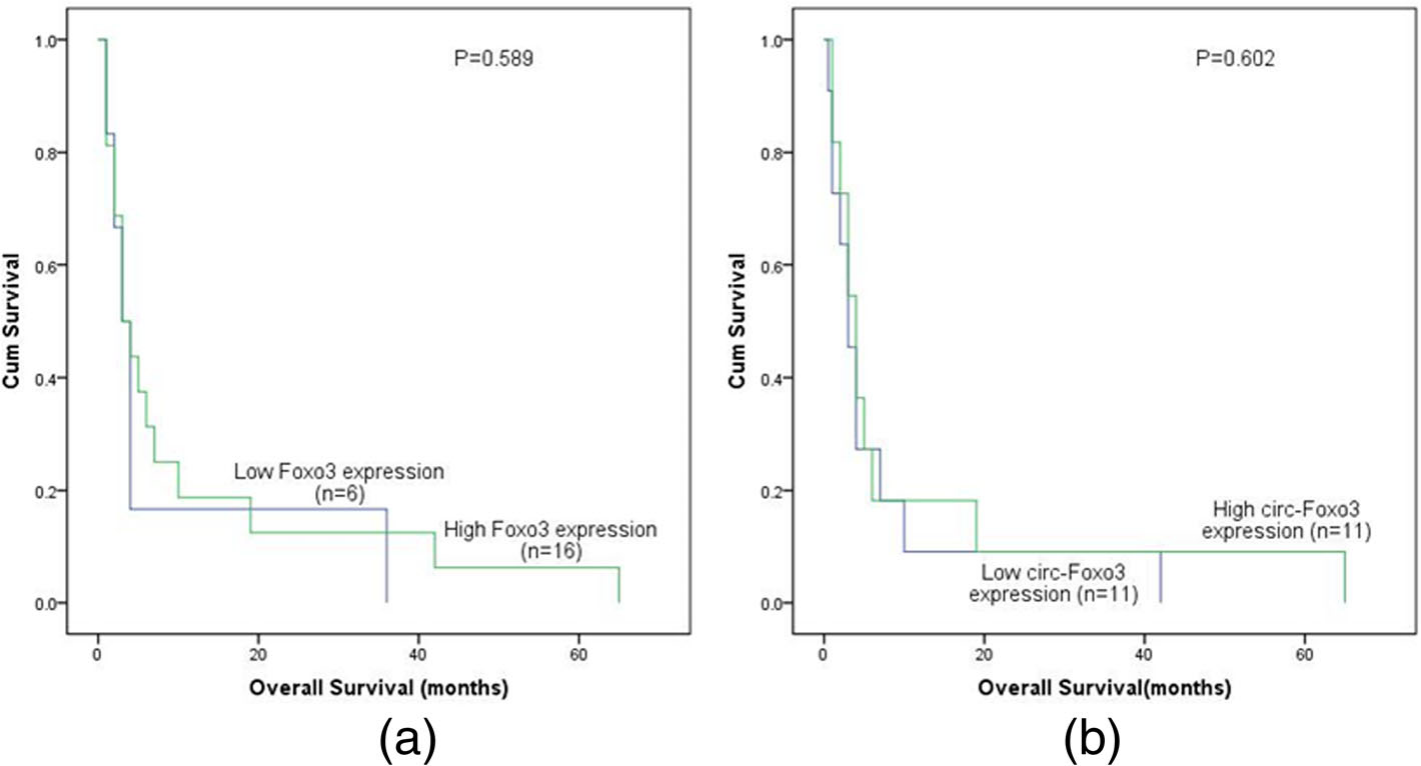
The impact of *Foxo3* and *circ-Foxo3* expression on overall survival time of the AML patients below 65 years and intermediate group. **a** *Foxo3* **b** *circ-Foxo3*

In our results, it showed that expression of *Foxo3* (*P* = 0.009) and *circ-Foxo3* (*P* = 0.04) was both down regulated in AML patients. Then we conducted Kaplan-meier survival analysis, we found that there was a tendency that *Foxo3* ^high^ group patients had longer survival time than *Foxo3* ^low^ group in these patients we tested, but it was not statistically significant (*P* = 0.192) (Fig. 4). As we mentioned above, AML patients can be assorted into different classifications according to different basis for grouping. In non-M3, non-favorable and normal karyotype group, *Foxo3* ^high^ group patients survived longer than *Foxo3* ^low^ group obviously (Fig. 5). Similarly, the LFS time was compared between *Foxo3* ^high^ and *Foxo3* ^low^ patients. The result showed that *Foxo3* ^high^ group patients had longer LFS time than that of *Foxo3* ^low^ group in normal karyotype, non-M3 and non-favorable risk group (Fig. 6). At last, Kaplan-Meier analysis was also conducted on intermediate patients below 65 years. But it showed no statistical significance (Fig. 7).

Kaplan-meier analysis was further performed, and it found out that there was no statistical significance correlation between *circ-Foxo3* expression and OS time of AML patients. Maybe it was due to the small amount of specimens we used in the experiments, so that the results could not represent general characteristics. In the future, we will collect more patient samples and test the level of *circ-Foxo3* expression. According to our current experimental results, we were unable to conclude that *circ-Foxo3* was a prognostic factor that affecting survival time of AML patients.

William W Du et al. discovered that, after treated with H_2_O_2_, Cisplatin and Doxorubicin, the expression of *circ-Foxo3* was up-regulated in cancer cell lines (66C14, 4 T1, MDA-MB-468, and MDA-MB-231) [20]. They also found that MB-231 cells transfected with siRNA-targeting *circ-Foxo3* could decrease *Foxo3* level [20]. Moreover, William W Du et al. discovered that ectopic *circ-Foxo3* could increase *Foxo3* level in MB-231 cells [20], and which means the expression of the *circ-Foxo3* and *Foxo3* was positively correlated. Not surprisingly, in our research, we found that same phenomenon in cell lines. The expression of *Foxo3* and *circ-Foxo3* were positively corrected not only in cell lines (K562, U937, NB4, SHI-1, and HEL) (R = 0.98, *P* < 0.0021) (Fig. 3), but also in AML patients (R = 0.63, *P* < 0.0001) (Fig. 3). Results showed that the expression of *Foxo3* in AML patients was significantly down-regulated. Previous studies revealed that up-regulated expression of *circ-Foxo3* triggered stress-induced apoptosis and inhibited tumor growth. In normal breast mammary tissues, higher circRNA was observed, and it appears to be inversely correlated with the risk-of-relapse proliferation score for proliferation genes in breast cancer [20]. It has been demonstrated that up-regulation of *Foxo3* can promote apoptosis by upregulated pro-apoptotic Bcl-2 family members (Bim), and leading to an increase of cell cycle protein levels in p27kip1 [25–27]. Xinbo et al. hold the view that *Foxo3* can promote apoptosis through activating pro-apoptotic proteins (like Bim and Bad), death receptor ligands (like Fas); tumor necrosis factor related apoptosis-inducing ligand and cyclin-dependent kinase inhibitors [28]. So we could infer that low level of *circ-Foxo3* down-regulated *Foxo3* and inhibited these pro-apoptotic factors so that patients with low level expression of *circ-Foxo3* have shorter survival time.

Besides, Fei et al. found that low level of *Foxo3* was reported to be related to chemotherapy resistance and associated with poor prognosis of ovarian cancer patients [22]. In fact, the recovery of *Foxo3* expression had been developed for some mechanism-based anticancer therapies [29, 30]. Furthermore, *Foxo3* was widely considered to be an important factor affecting the efficacy of various chemotherapy drugs. Coincidently, in our study, patients with high level of *Foxo3* had better prognosis than those with low level. On clinical, the chemotherapy drugs that commonly used were Daunorubicin, Cytarabine, Thioguanine, Harringtonine, Mitoxantrone, etc. The patients, in our medical center, were all treated by chemotherapy. Maybe patients with high level of *Foxo3* were more sensitive to these drugs. After standard chemotherapy, patients with high level of *Foxo3* expression often lived longer than those with low level. We may infer that lower *Foxo3* expression contribute patients to drug resistance. In order to validate our conjecture, we will design and complete more experiments in the future. Combined with above consequence, we may speculate that AML patients with high expression of *Foxo3* were sensitivity towards current chemotherapy. Our experimental results were exactly in line with the above results.

Moreover, not only circ-RNA and linear gene can affect the outcome of AML cancers, but also micro RNA can make contributions. Some reported that *Foxo* family transcripts were firmly regulated by the microRNA networks in cancer progression and metastasis [31–33]. For example, reduced miR-215 expression predicts poor prognosis in patients with acute myeloid leukemia [34]. As we mentioned above, circular RNAs can sponge microRNA, and serves as a competitive inhibitor for microRNA [15]. After analyzing our results of experiments, we found that *Foxo3* and *circ-Foxo3* gene were both down-regulated and positively corrected in AML patients. Combined with the existing theory, we speculated that the down-regulated *circ-Foxo3* could release some relevant mircoRNA, and contribute to the low expression of *Foxo3* correspondingly; so that these pro-apoptotic factors would be suppressed. At last, patients with low level of *circ-Foxo3* and *Foxo3* would have adverse prognosis.

We had tested some miRNA in AML, but we have not yet found a miRNA to be correlated with *circ-Foxo3* and *Foxo3*. Based on the results, we will study some other microRNAs and analysis the relation of *circ-Foxo3*, microRNA and *Foxo3* gene. In the future, we will conduct more researches in detail to verify current conclusions. We hope our effort could make sense for diagnosis and therapy for AML patients.

## Conclusion

In summary, we could conclude that *circ-Foxo3* and *Foxo3* were negatively correlated and low level of *Foxo3* gene was a frequent molecular event which leads to adverse prognosis of AML patients.

## Data Availability

The datasets used and/or analyzed during the current study available from the corresponding author on reasonable request.

## Abbreviations

AML: Acute myeloid leukemia
BM: Bone Marrow
BMMC: The mononuclear cells from bone marrow
circRNA: Circular RNA (
CVD: Cardiovascular Disease
FAB: The France- America– British
HB: Hemoglobin
OS: Overall Survival
PLT: Platelets
ROC: Receiver operating characteristic curve
WBC: White Blood Cells
WHO: World Health Organization criteria
